# Assessing the accessibility of HIV care packages among tuberculosis patients in the Northwest Region, Cameroon

**DOI:** 10.1186/1471-2458-10-129

**Published:** 2010-03-12

**Authors:** Nwarbébé Barnabas Njozing, San Sebastian Miguel, Pius Muffih Tih, Anna-Karin Hurtig

**Affiliations:** 1 St Mary Soledad Catholic Hospital, Mankon, Bamenda, PO Box 157, Cameroon; 2 Department of Public Health and Clinical Medicine, Unit of Epidemiology and Global Health, Umeå University, Umeå, 901 85, Sweden; 3 Swedish Research School for Global Health, Umeå University, 901 85, Umeå, Sweden; 4 Cameroon Baptist Convention Health Board, Nkwen, Bamenda, PO Box 1, Cameroon

## Abstract

**Background:**

Tuberculosis (TB) and human immunodeficiency virus (HIV) co-infection is a major source of morbidity and mortality globally. The World Health Organization (WHO) has recommended that HIV counselling and testing be offered routinely to TB patients in order to increase access to HIV care packages. We assessed the uptake of provider-initiated testing and counselling (PITC), antiretroviral (ART) and co-trimoxazole preventive therapies (CPT) among TB patients in the Northwest Region, Cameroon.

**Methods:**

A retrospective cohort study using TB registers in 4 TB/HIV treatment centres (1 public and 3 faith-based) for patients diagnosed with TB between January 2006 and December 2007 to identify predictors of the outcomes; HIV testing/serostatus, ART and CPT enrolment and factors that influenced their enrolment between public and faith-based hospitals.

**Results:**

A total of 2270 TB patients were registered and offered pre-HIV test counselling; 2150 (94.7%) accepted the offer of a test. The rate of acceptance was significantly higher among patients in the public hospital compared to those in the faith-based hospitals (crude OR 1.97; 95% CI 1.33 - 2.92) and (adjusted OR 1.92; 95% CI 1.24 - 2.97). HIV prevalence was 68.5% (1473/2150). Independent predictors of HIV-seropositivity emerged as: females, age groups 15-29, 30-44 and 45-59 years, rural residence, previously treated TB and smear-negative pulmonary TB. ART uptake was 50.3% (614/1220) with 17.2% (253/1473) of missing records. Independent predictors of ART uptake were: previously treated TB and extra pulmonary TB. Finally, CPT uptake was 47.0% (524/1114) with 24% (590/1114) of missing records. Independent predictors of CPT uptake were: faith-based hospitals and female sex.

**Conclusion:**

PITC services are apparently well integrated into the TB programme as demonstrated by the high testing rate. The main challenges include improving access to ART and CPT among TB patients and proper reporting and monitoring of programme activities.

## Background

Globally, tuberculosis (TB) morbidity and mortality is partly attributed to the co-infection with human immunodeficiency virus (HIV). In 2007, there were an estimated 13.7 million prevalent TB cases and 9.27 million new cases out of which 4.1 million (44%) were smear-positive cases. Africa accounted for 31% of the new TB cases [1]. Similarly, 33.2 million HIV prevalent and 2.5 million incident cases were recorded worldwide in 2007. Sub-Saharan Africa alone accounted for 22.5 million of the prevalent cases and 1.7 million of the incident cases [2]. Among the 9.27 million incident TB cases, 1.37 million (14.8%) were HIV-positive and Africa accounted for 79% of these cases. There were an estimated 1.32 million deaths from HIV-negative TB patients and 456 000 TB deaths among HIV-positive patients. Deaths from co-infected patients accounted for 23% of the estimated 2 million HIV deaths [1].

The increasing co-infection prompted the World Health Organization (WHO) to launch the "ProTEST" initiative as a strategy to reduce the burden of HIV-related TB requiring collaboration between HIV and TB programmes [3]. It promoted voluntary counselling and testing as an entry point to access a range of TB/HIV prevention and care interventions, especially in areas with high HIV prevalence. HIV counselling and testing has since been recommended routinely to all TB patients [4].

Currently, the concept of provider-initiated HIV testing and counselling (PITC) (which refers to HIV counselling and testing recommended by health care providers to people attending health facilities as a standard component of medical care) has been advocated. This presents an opportunity to ensure that HIV is more systematically diagnosed in health care services in order to facilitate patient access to needed HIV prevention, treatment, care and support services [5]. Through this strategy, TB treatment centres have been shown to serve as important fora for identifying patients with HIV infection [6-8] and uptake of HIV testing among TB patients has been impressive. Between 2002 and 2007, testing for HIV increased from 21 806 (0.5%) to 1 million (16%) among the notified TB cases globally. In Africa, 491 755 TB patients were tested for HIV in 2007 and this represented 36% of all notified cases compared to 22% in 2006. Meanwhile enrolment on antiretroviral therapy (ART) and cotrimoxazole preventive therapy (CPT) has also been growing steadily in absolute terms. In 2007, 90 000 TB patients were on ART and 200 000 were on CPT. However, this has been accompanied by a fall in the percentage of TB patients whom are diagnosed with HIV and whom are enrolled on ART and CPT (from 40% in 2006 to 34% in 2007 and 77% to 63% respectively). In Africa, enrolment reached 33% for ART and 66% for CPT [1].

In Cameroon, the National AIDS Control Committee was created in 1986 and later, in 1987, the National AIDS Control Programme (NACP) was launched. The national response to HIV/AIDS has received political support and since 1999 the fight against HIV/AIDS is one of the national priorities. The Government drafted the first National Strategic Plan against AIDS for 2000-2005 which aimed essentially to prevent new infections by promoting voluntary counselling, treating infected persons and reducing the cost of treatment, promoting research and preventing mother-to-child transmission of HIV. The second plan for 2006-2010 is multi-sector based, decentralized and involves the civil society, private sector and grass-root communities. The second plan comprises 6 strategic axes: universal access to HIV prevention, universal access to treatment and care among children and adults living with HIV/AIDS, protection and assistance to orphans and vulnerable children, involving actors in the fight against HIV/AIDS, epidemiological surveillance, promoting research and reinforcing coordination, partnership, monitoring/evaluation [9]. The national HIV-seroprevalence was 5.4% in 2004 [10] and there are 95 sites providing ART treatment nationwide. Since May 2007, ART and CPT have been provided free of charge to those eligible. At the end December 2008, there were 153 185 HIV patients eligible for ART (88 678 females [57.9%] and 64 506 males [42.1%]). A total of 59 960 (39.1%) of these patients were enrolled on ART (40 357 females [67.3%] and 19 603 males [32.7%]) [9].

The National TB Control Programme (NTCP) was launched in 1996 and, in 2002, it was recognized as a priority programme by the Ministry of Public Health. The NTCP is organized according to three levels of intervention: central, regional and peripheral. The Ministry of Public Health is responsible for the organization and implementation of the programme at the central level and provides the annual budget and permanent financial support. The central level comprises the National Tuberculosis Control Committee (which defines the general objectives of the programme), the Consultative Scientific Committee, the Central Technical Group (which is the executive organ), the Tuberculosis National Reference Laboratory and the Chest Service of the Jamot Hospital (which is a third referral level in matters of TB control in the country). The regional level is under the authority of the Regional Delegate of Public Health and has as its mission the organization, coordination, follow-up and evaluation of the fight against TB in the region. The peripheral level comprises the health districts that make up the structure on which the TB programme is built and participates in TB case finding and treatment, as well as the keeping of the TB register. The specific objectives of the NTCP are to cure at least 85% of detected cases of sputum-smear positive pulmonary TB (PTB), to detect 70% of existing cases of sputum-smear positive PTB patients, and to protect through BCG vaccination at least 80% of children born each year [11].

The country is implementing the Directly Observed Treatment, Short-Course (DOTS) strategy with 100% coverage [1]. There are 217 TB diagnostic and treatment centres in 142 health districts nationwide. The case detection rate from DOTS services in 2007 for all new cases was 65% and 91% for new smear-positive cases [1]. The case notification rate has been increasing steadily since 2000 from 33/100 000 population/year to 130 in 2007. A total of 36 088 prevalent and 35 556 incident TB cases were recorded in 2007. Among the 2006 cohort (13 811 patients) of smear-positive patients, a 64% cure rate was recorded with a 74% treatment success rate, 6% death rate and 13% default rate [1]. Collaboration with the AIDS Control Programme began in 2004 and HIV counselling and testing are now routine and have been free for all TB patients since 2006. Since 2006, the number of TB patients tested for HIV has increased from 8 637 to 11 825 in 2007 and to 16 144 in 2008 with corresponding co-infection rates of 38.9%, 43.8% and 40.4% respectively in the adult population [9].

In 2004, the Northwest Region (NWR), one of the 10 regions in the country, had the highest HIV-seroprevalence of 8.7% (11.9% amongst females compared to 5.2% amongst males [10]) and HIV-related TB still remains a public health priority in the region. Since 1990, when multi-party politics was introduced in the country, there have been several anti-government demonstrations especially in the NWR which is the stronghold of the major opposition party in the country: the social democratic front (SDF). This has led to riots and civil unrest with frequent clashes between law enforcement officials and the public with several reported incidences of sexual harassment and rape which could account partly for the high HIV prevalence in the region.

Although TB/HIV collaborative activities began in 2004, programmatic changes involved re-designing TB registers to capture uptake of HIV testing, ART and CPT services. These registers went operational in all TB diagnostic and treatment centres in January 2006. The staffs in the centres were also trained in TB diagnosis and treatment, as well as record keeping and reporting. This study assessed the uptake of PITC, ART and CPT services and also factors that influenced their uptake among TB patients in selected treatment facilities within the NWR between January 2006 and December 2007 when TB/HIV collaborative activities became operational.

## Methods

### Study setting

Cameroon's population is approximately 18.4 million with an estimated land area of 475 440 km^2^. There are 10 regions and this study was carried out in the NWR, which has a population of over 1.8 million and is comprised of 7 divisions; the capital, Bamenda, has an urban population of over 300 000. There are 13 health districts with 1 regional hospital, 19 district hospitals, and 106 assimilated district hospitals and health centres [10]. In addition, there are private and faith-based hospitals with primary health centres.

Four hospitals out of 10 providing comprehensive TB/HIV treatment and support services in the NWR were purposively selected because of: i) their accessibility; ii) patient load; iii) diversity of patients since they serve both rural and urban populations; iv) similarity in the services provided since they act as referral centres in the region; and v) possibility to evaluate the services between public and faith-based settings because of perceived differences amongst the population with regards to the quality of patient care, user friendliness and cost in accessing treatment. These included 1 public hospital (Bamenda Regional Hospital) and 3 faith-based hospitals: Banso Baptist Hospital, Mbingo Baptist Hospital, and Njinikom Catholic Hospital.

### Tuberculosis services

TB diagnosis, treatment, follow-up and documentation in these facilities are intended to follow the national guidelines [11]. Diagnosis is by sputum microscopy which costs 1000 FCFA (2.4 US$) during the entire TB treatment. Patients with at least 1 sputum smear positive for acid-fast bacilli are classified smear-positive PTB. In cases with negative smears, diagnosis is made clinically and/or by chest x-ray findings consistent with PTB since sputum culture is not routinely performed because of cost and inadequate human resources. Diagnosis of extra-pulmonary TB (EPTB) is based on clinical and/or radiological findings, biopsies and laboratory examination of aspirates from affected areas.

Anti-TB drugs have been provided free of charge since 2004 and treatment of newly diagnosed PTB/EPTB lasts 6 months, comprising an initial 2-month intensive phase with a fixed dose combination of Rifampicin, Isoniazid, Pyrazinamide and Ethambutol with an initial 2 weeks of hospitalisation; this is followed by a 4-month continuous phase of daily Rifampicin and Isoniazid. Diagnosis and treatment are made by trained medical officers but follow-up and documentation are performed by trained nurses.

### Counselling services

All diagnosed TB cases are counselled for HIV by trained counsellors within the TB unit using the 'opt-out' approach, where patients reserve the right to accept or refuse testing for HIV without reprisal from providers. Post-test counselling is offered to all who accept testing regardless of the outcome, support services to HIV-positive cases like the chaplaincy department and HIV support groups are also made available. Based on the national treatment guidelines, ART is initiated in the following scenarios: i) if the CD4-count is <200 cells/mm^3 ^between 2-8 weeks after commencing anti-TB treatment; ii) if the CD4-count is between 200-350 cells/mm^3 ^after 8 weeks of anti-TB treatment; iii) if the CD4-count is above 350 cells/mm^3^, ART is differed and the patient is re-evaluated after 8 weeks and at the end of anti-TB treatment; and iv) in the absence of CD4-count, if the total lymphocyte count is <1200 cells/mm^3^, ART is introduced between 2-8 weeks of anti-TB treatment [12].

### Data sources and analysis

Data were obtained from TB treatment registers in the study hospitals for patients diagnosed between January 2006 and December 2007. Register data in the treatment centres are collated and forwarded to the Ministry of Public Health. Basic socio-demographic information, TB profile and HIV services were obtained. HIV treatment registers were also reviewed for information on ART for cases not documented in TB registers.

Data were entered using Microsoft Excel™ and analyzed using Epi Info™ version 3.4.3. Descriptive statistics of patients were performed and main outcome variables (HIV testing and status, ART and CPT uptake) were dichotomized and potential factors associated with the outcomes were tested individually by univariate analysis. Logistic regression analysis was performed having controlled for other variables to identify independent predictors using odds ratio (OR), 95% confidence interval (95% CI) and p-values less than 0.05 were considered statistically significant.

### Ethical approval

Ethical approval for the study was obtained from the Delegation of Public Health for the NWR (N° 401/NWP/PDPH/08), Internal Review Board of the Regional Hospital, Bamenda and, the Cameroon Baptist Convention Health Board Institutional Review Board (IRB 2007-09). Informed consent was also obtained from the Regional coordinator of TB and TB coordinators/nurses in the study hospitals. They were assured that all information in the TB/HIV registers would be treated with strict confidentiality. During data entry, only the patients' serial numbers in the records were collected to ensure anonymity and the data was password-protected.

## Results

### Patient Characteristics

A total of 2270 TB patients were treated between January 2006 and December 2007. The mean age of the patients was 34 years (range 1 month - 88 years). The detailed patients' characteristics are presented in Table 1.

**Table 1:** TB patients' characteristics by study hospitals 2006-2007 (n = 2270)

Variable	ALL	BBH†	MBH	BRH	NJH
	n (%)	468 (20.6)	564 (24.8)	1063 (46.8)	175 (7.7)
**Sex**					
Male	1163 (51.2)	255 (54.5)	315 (55.9)	509 (47.9)	84 (48.0)
Female	1107 (48.8)	213 (45.5)	249 (44.1)	554 (52.1)	91 (52.0)
**Age Group (years)**					
0-14	98 (4.3)	20 (4.3)	29 (5.1)	44 (4.1)	5 (2.9)
15-29	772 (34.0)	164 (35.0)	197 (34.9)	343 (32.3)	68 (38.9)
30-44	971 (42.8)	193 (41.2)	232 (41.1)	464 (43.7)	82 (46.9)
45-59	308 (13.6)	61 (13.0)	75 (13.3)	158 (14.9)	14 (8.0)
≥ 60	121 (5.3)	30 (6.4)	31 (5.5)	54 (5.1)	6 (3.4)
**Residence**					
Rural	1180 (52.0)	404 (86.3)	302 (53.5)	323 (30.4)	151 (86.3)
Urban	1090 (48.0)	64 (13.7)	262 (46.5)	740 (69.6)	24 (13.7)
**TB Type**					
SPPTB	1369 (60.3)	361 (77.1)	330 (58.5)	582 (54.8)	96 (54.9)
SNPTB	534 (23.5)	38 (8.1)	130 (23.0)	290 (27.3)	76 (43.4)
EPTB	367 (16.2)	69 (14.7)	104 (18.4)	191 (18.0)	3 (1.7)
**TB Category**					
New	2081 (91.7)	458 (97.9)	543 (96.3)	920 (86.5)	160 (91.4)
Others*	189 (8.3)	10 (2.1)	21 (3.7)	143 (13.5)	15 (8.6)
**Treatment Outcome**					
Treatment Success^¶^	1602 (70.6)	354 (75.7)	385 (68.2)	751 (70.7)	112 (64.0)
Cured	855 (37.7)	240 (51.3)	161 (28.5)	406 (38.2)	48 (27.4)
Completed treatment	747 (32.9)	114 (24.4)	224 (39.7)	345 (32.5)	64 (36.6)
Died	297 (13.1)	70 (15.0)	60 (10.6)	136 (12.8)	31 (17.7)
Treatment failure	13 (0.6)	5 (1.1)	5 (0.9)	3 (0.3)	0 (0)
Treatment default	111 (4.9)	21 (4.5)	31 (5.5)	40 (3.8)	19 (10.9)
Transferred out	247 (10.9)	18 (3.8)	83 (14.7)	133 (12.5)	13 (7.4)

† BBH (Banso Baptist Hospital), MBH (Mbingo Baptist Hospital), BRH (Bamenda Regional Hospital), NJH (Njinikom Hospital)

*Others - Includes treatment failure, treatment default, transferred in from another facility and relapse cases

¶ Treatment Success = Cured + Completed treatment

### HIV Testing

**Figure 1:**
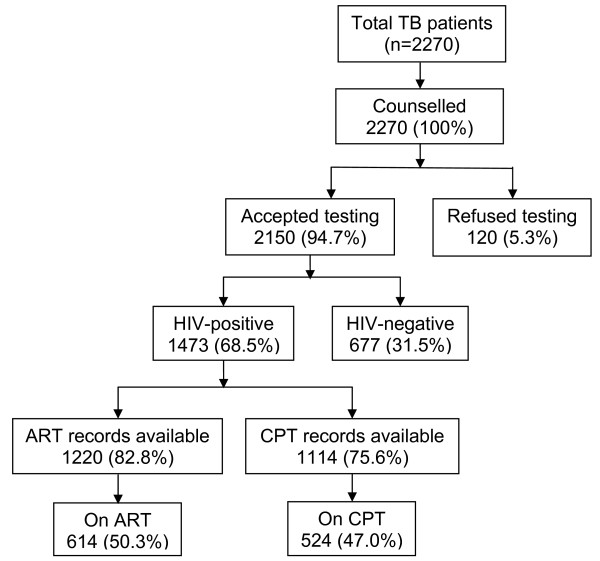
**A schematic representation of HIV diagnostic processes and outcomes among TB patients**.

All 2270 patients were offered pre-HIV test counselling and the outcomes are presented in Figure 1. In the public hospital, 96.4% (1025/1063) patients were tested compared to 93.2% (1125/1207) in faith-based hospitals. Among all the variables considered, testing was significant only in the public hospital compared to the faith-based hospitals (crude OR 1.97; 95% CI 1.33 - 2.92) and (adjusted OR 1.92; 95% CI 1.24 - 2.97) (Data not shown).

### HIV Seroprevalence

A total of 2150 patients were tested for HIV and the outcomes are presented in Figure 1. In the public hospital, 76.0% (779/1025) of patients tested positive compared to 61.7% (694/1125) in the faith-based hospitals. Patients in the public hospital were more likely to be HIV-positive compared to those in faith-based hospitals (crude OR 1.97; 95% CI 1.63 - 2.37) and (adjusted OR 2.08; 95% CI 1.66 - 2.61). Females, age groups 15-29, 30-44 and 45-59 years, patients from rural areas, previously treated TB and SNPTB emerged as independent predictors of HIV-seropositivity in multivariate analysis (Table 2).

**Table 2:** Logistic regression analysis of HIV status with respect to potential predictors

Variable	HIV Positive^#^	Univariate Analysis			Multivariate Analysis		
	N (%)	OR^a^	95% CI^b^	P-value	OR^c^	95% CI	P-value
**All**	1473 (68.5)						
							
**Health Facility**							
Faith-based	694 (61.7)	1	-	-	1	-	-
Public	779 (76.0)	**1.97**	**(1.63 - 2.37)**	**0.000**	**2.08**	**(1.66 - 2.61)**	**0.000^d^**
							
**Sex**							
Male	700 (64.0)	1	-	-	1	-	-
Female	773 (73.1)	**1.53**	**(1.27 - 1.84)**	**0.000**	**1.78**	**(1.45 - 2.19)**	**0.000**
							
**Age Group**							
0-14	44 (48.4)	1	-	-	1	-	-
15-29	428 (58.5)	1.51	(0.98 - 2.34)	0.065	**2.16**	**(1.33 - 3.50)**	**0.002**
30-44	754 (81.3)	**4.66**	**(2.99 - 7.25)**	**0.000**	**7.28**	**(4.47 - 11.87)**	**0.000**
45-59	208 (72.0)	**2.74**	**(1.69 - 4.45)**	**0.000**	**3.90**	**(2.31 - 6.60)**	**0.000**
≥ 60	39 (34.8)	0.57	(0.32 - 1.00)	0.052	0.70	(0.38 - 1.28)	0.249
							
**Residence**							
Rural	756 (68.0)	1	-	-	1	-	-
Urban	717 (69.0)	1.05	(0.87 - 1.25)	0.631	**0.72**	**(0.58 - 0.89)**	**0.003**
							
**Category of Patients**							
Others*	143 (79.0)	1	-	-	1	-	-
New	1330 (67.5)	**0.55**	**(0.38 - 0.80)**	**0.002**	**0.58**	**(0.39 - 0.87)**	**0.008**
							
**TB Type**							
EPTB	206 (60.2)	1	-	-	1	-	-
SNPTB	417 (81.3)	**2.87**	**(2.10 - 3.91)**	**0.000**	**3.03**	**(2.16 - 4.25)**	**0.000**
SPPTB	850 (65.6)	1.26	(0.99 - 1.61)	0.064	1.13	(0.86 - 1.49)	0.387

a- crude odds ratio

b- 95% confidence interval

c- adjusted odds ratio

d- significant values are highlighted

*Others - Includes treatment failure, treatment default, transferred in from another facility and relapse cases

# Denominator is the total number of patients tested for HIV (2150)

### Antiretroviral Therapy Uptake

Records on ART were available for 1220 of 1473 HIV-positive TB patients and 50.3% (614) were enrolled on ART; 52.9% (408/771) in the public and 45.9% (206/449) in faith-based hospitals). Enrolment was significantly higher in the public hospital compared to the faith-based hospitals in univariate analysis (crude OR 1.33; 95% CI 1.05 - 1.67). Previously treated TB and EPTB emerged as independent predictors of ART enrolment in multivariate analysis (Table 3).

**Table 3:** Logistic regression analysis of ART uptake with respect to potential predictors

Variable	ART^#^	Univariate Analysis			Multivariate Analysis		
	N (%)	OR^a^	95% CI^b^	P-value	OR^c^	95% CI	P-value
**All**	614 (50.3)						
							
**Health Facility**							
Faith-based	206 (45.9)	1	-	-	1	-	-
Public	408 (52.9)	**1.33**	**(1.05 - 1.67)**	**0.018**	1.13	(0.87 - 1.46)	0.371
							
**Sex**							
Male	281 (49.6)	1	-	-	1	-	-
Female	333 (51.0)	1.06	(0.85 - 1.33)	0.617	1.12	(0.88 - 1.42)	0.356
							
**Age Group**							
0-14	17 (44.7)	1	-	-	1	-	-
15-29	156 (44.7)	1.00	(0.51 - 1.96)	0.997	1.26	(0.63 - 2.53)	0.513
30-44	322 (52.1)	1.34	(0.70 - 2.60)	0.379	1.64	(0.84 - 3.22)	0.149
45-59	101 (56.4)	1.60	(0.79 - 3.25)	0.191	1.93	(0.95 - 3.95)	0.071
≥ 60	18 (50.0)	1.24	(0.50 - 3.08)	0.651	1.32	(0.52 - 3.32)	0.559
							
**Residence**							
Rural	270 (46.6)	1	-	-	1	-	-
Urban	344 (53.8)	**1.33**	**(1.07 - 1.67)**	**0.012**	1.20	(0.94 - 1.54)	0.144
							
**Category of Patients**							
Others*	75 (57.7)	1	-	-	1	-	-
New	539 (49.4)	0.72	(0.52 - 1.04)	0.077	**0.63**	**(0.43 - 0.93)**	**0.019^d^**
							
**TB Type**							
EPTB	107 (56.0)	1	-	-	1	-	-
SNPTB	212 (55.6)	0.98	(0.69 - 1.40)	0.931	1.02	(0.72 - 1.46)	0.899
SPPTB	295 (45.5)	0.66	(0.47 - 0.91)	0.011	**0.65**	**(0.47 - 0.92)**	**0.013**

a- crude odds ratio

b- 95% confidence interval

c- adjusted odds ratio

d- significant values are highlighted

*Others - Includes treatment failure, treatment default, transferred in from another facility and relapse cases

# Denominator is the total number of HIV-positive patients with ART records available (1220)

### Co-trimoxazole Preventive Therapy Uptake

Records on CPT were available for 1114 of 1473 HIV-positive patients and 47.0% (524) were enrolled on CPT: 35.2% (241/684) in the public hospital and 65.8% (283/430) in the faith-based hospitals. Enrolment was significantly lower in the public hospital compared to the faith-based hospitals (crude OR 0.28; 95% CI 0.22 - 0.36) and (adjusted OR 0.29; 95% CI 0.22 - 0.38). Female sex also emerged as an independent predictor of CPT enrolment in multivariate analysis (Table 4).

**Table 4:** Logistic regression analysis of CPT uptake with respect to potential predictors

Variable	CPT^#^	Univariate Analysis			Multivariate Analysis		
	N (%)	OR^a^	95% CI^b^	P-value	OR^c^	95% CI	P-value
**All**	524 (47.0)						
							
**Health Facility**							
Faith-based	283 (65.8)	1	-	-	1	-	-
Public	241 (35.2)	**0.28**	**(0.22 - 0.36)**	**0.000**	**0.29**	**(0.22 - 0.38)**	**0.000^d^**
							
**Sex**							
Male	231 (45.0)	1	-	-	1	-	-
Female	293 (48.8)	1.16	(0.92 - 1.47)	0.215	**1.32**	**(1.02 - 1.71)**	**0.036**
							
**Age Group**							
0-14	13 (35.1)	1	-	-	1	-	-
15-29	148 (45.7)	1.55	(0.76 - 3.16)	0.224	0.89	(0.42 - 1.90)	0.766
30-44	280 (49.4)	1.80	(0.90 - 3.61)	0.097	1.26	(0.60 - 2.60)	0.542
45-59	73 (46.5)	1.60	(0.76 - 3.38)	0.213	1.20	(0.55 - 2.61)	0.649
≥ 60	10 (34.5)	0.97	(0.35 - 2.70)	0.956	0.74	(0.26 - 2.15)	0.583
							
**Residence**							
Rural	290 (54.4)	1	-	-	1	-	-
Urban	234 (40.3)	**0.57**	**(0.45 - 0.72)**	**0.000**	0.86	(0.65 - 1.12)	0.256
							
**Category of Patients**							
Others*	57 (47.5)	1	-	-	1	-	-
New	467 (47.0)	0.98	(0.67 - 1.43)	0.915	0.77	(0.51 - 1.17)	0.223
							
**TB Type**							
EPTB	74 (42.3)	1	-	-	1	-	-
SNPTB	145 (42.9)	1.03	(0.71 - 1.48)	0.894	0.83	(0.65 - 1.12)	0.256
SPPTB	305 (50.7)	**1.41**	**(1.00 - 1.98)**	**0.049**	0.83	(0.56 - 1.23)	0.350

a- crude odds ratio

b- 95% confidence interval

c- adjusted odds ratio

d- significant values are highlighted

*Others - Includes treatment failure, treatment default, transferred in from another facility and relapse cases

# Denominator is the total number of HIV-positive patients with CPT records available (1114)

## Discussion

Our study revealed that overall uptake of PITC among TB patients in the region is high (94.7%). This high testing rate might be attributed to the fact that HIV/AIDS is now seen as a "normal" disease for which there is free, life-saving treatment available. It might also be due to the influence of healthcare providers, since it is rare for patients to object to decisions from health authorities. High testing rates have also been reported in other studies in Africa [13-15]. Testing rates were slightly higher in the public hospital compared to the faith-based hospitals but other variables assessed in the study showed no significant associations with testing rates. Further research is needed to identify the reasons for the underlying structural differences in testing between treatment facilities and to evaluate the impact of other socio-economic factors on testing which we were unable to assess because they have not been documented routinely in TB registers.

The study demonstrated a 68.5% HIV prevalence among TB patients, similar to other studies in Africa [14-16] but higher than the national figures for 2006 (38.9%) and 2007 (43.8%) [9]. These discrepancies could be attributed to the high HIV prevalence in the NWR and because of improved monitoring and reporting services compared to the pre-TB/HIV collaborative era. HIV-seroprevalence was higher among females, 73.1% (773/1057) consistent with results of the 2004 country Demographic Health survey [10] and findings in other studies [15-17]. The 15-59 years age group, which is the productive population, was the group most affected by the epidemic. One of the strategies of the NACP is to provide universal access to HIV prevention as evidenced by the numerous HIV sensitization and prevention campaigns nationwide. However, future efforts should specifically address the needs of the adult population, especially females and the rural communities who are particularly disadvantaged socio-economically and thus more vulnerable to the infection.

Our study revealed a 50% (614/1220) uptake of ART among TB patients who had their HIV treatment history documented (82.8%). Assuming all co-infected patients are eligible for ART in the absence of CD4-count [18], then the true coverage of ART among TB patients would be 41.7% (614/1473). These figures are relatively higher than findings in Malawian studies (13% and 16%) [19,20]. Plausible explanations for the findings in our study could be the scale-up and decentralisation of ART services in the country [21], increasing awareness of the benefits of ART which has encouraged testing for HIV, reduction in the cost of the CD4-count test to increase ART eligibility, and training/increasing the number of HIV treatment officers within the facilities. Despite the relatively high uptake of ART in the region compared to other studies, our study revealed lapses in data reporting. Seventeen percent (253/1473) of ART data were not documented in the registers, which reflects the operational challenges in establishing mechanisms for, and implementing, a sound monitoring, reporting, and evaluation of programme activities; this calls for a rigorous reporting system, an issue which could be addressed with the use of electronic medical records (EMR). Despite its challenges (which include setting up and maintaining the system, training of local staff in data entry and management and ensuring a stable power supply to minimise data loss) EMR have been used successfully to support HIV/TB treatment in resource-limited settings [22,23], improving not only patient and programme monitoring, reporting and evaluation, but also management of drug supplies. However, any consideration of such systems will have to reflect the local constraints and adapt to the local needs. Our study also revealed that previously treated TB patients were more likely to be on ART compared to newly diagnosed TB patients. Recently, the SAPIT study in South Africa [24] showed that mortality among TB/HIV co-infected patients can be reduced by 55% if ART is provided concomitantly with TB treatment. Therefore, to ensure a sustained scale-up of ART and reduce morbidity and mortality associated with the co-infection, concomitant treatment with ART should be a priority. However, this should be guided by their CD4-count result which underlines the need for a proper monitoring system. Moreover, making CD4-count tests free of charge would increase eligibility and improve ART uptake in the long run.

We also demonstrated a low uptake of CPT (47.0%) among co-infected patients with 24% (359/1473) of CPT records missing. Assuming all HIV-positive patients are eligible for CPT, then 36% (524/1473) of the TB patients were on CPT. Although uptake was better in the faith-based hospitals (65.8%) compared to the public hospital (35.2%), these figures are lower than results from studies in Malawi with uptakes above 90% [13,15]. Considering the fact that CPT is a simple intervention with less stringent eligibility criteria compared to ART, a higher uptake compared to ART could have reasonably been expected. The habit of clinicians tending to place more emphasis on ART since it is regarded as life-saving while neglecting CPT might be an explanation for this finding. Besides, probably due to multitasking in treatment centres, there might also be a tendency for staff to neglect documenting CPT activities. Incidences of inadequate supplies and rupture of CPT stocks have been reported and patients have been required to procure treatment personally and this might be another explanation for the low enrolment. However, it was encouraging to observe a higher CPT enrolment among females. It is common that African women are financially dependent on their partners and, because of this, there is apprehension about fair access to HIV services [20]. Studies in Africa have demonstrated that CPT reduces morbidity and mortality in TB/HIV co-infection [13,25,26]. It is essential for providers to be reminded of the importance of providing these simple but important interventions to HIV-infected patients and to vigorously monitor and report programme activities. Further research is also required to explore the reasons for the above finding and address the operational challenges.

Limitations in our study included the fact that the study sites were purposively selected and the figures obtained may not be a true reflection of the entire region. However, our samples were accredited treatment centres and also served as referral centres in the region and received a diversity of patients with characteristics similar to those in other centres. ART and CPT services should be recorded according to the national guidelines, but registers were not complete. Missing records in TB registers were searched in HIV registers by either comparing names on TB registers or checking for patients on Efavirenz^®^-based ART combinations to identify TB patients. This may have affected the accuracy and completeness of the data and underestimated the rates. Documentation of CD4-count results was not routinely performed and made it problematic to correctly assess ART eligibility. It is difficult therefore to assess if the 50% ART enrolment observed was due to inaccessibility to CD4-counts or because of a low proportion of TB patients being eligible for ART. Besides, ART uptake is time dependent and our model did not take this into account; this might have also affected our results. Moreover, the predictors in our model were patient-based and did not take into consideration the health sector factors like supply-oriented issues that might have affected uptake of these services. Our study assessed uptake of HIV services in TB patients shortly after implementation of collaborative activities; carrying out the study during this period might potentially have distorted the findings as operational challenges during the implementation phase may well have improved over time. Finally, it is unknown if the high uptake of PITC was influenced or based on voluntary and informed choices. A qualitative study to explore the counselling services would shed more light on this.

## Conclusions

Our study demonstrated that PITC services are apparently well integrated into the TB programme in the region as evidenced by the high uptake of testing. This high uptake should translate into improved access to ART and CPT and ensure concomitant treatment with anti-TB drugs in those eligible for ART. Another challenge is ensuring the free availability to all TB patients of CD4 tests, other pre-therapeutic investigations associated with HIV care, and medical consultations; such availability will increase ART eligibility and ensure sustainability of the scale-up of HIV services. As collaborative activities are strengthened nationwide, staff capacity building in ART treatment and the importance of managing HIV opportunistic infections including the provision of CPT should be a priority. Finally, while stressing the need for an efficient documentation of programme activities in treatment centres, the use of EMR will, despite its challenges, inevitably improve HIV/TB treatment, as well as the reporting and monitoring of programme activities.

## Competing interests

The authors declare that they have no competing interests.

## Authors' contributions

All authors contributed to the paper. NBN, AKH and MSS conceptualized and designed the study; NBN and PMT were responsible for the organization and collection of data; NBN performed the analysis which was interpreted by NBN, MSS and AKH; NBN drafted the manuscript with substantial revisions from all the authors. All authors read and approved the final version of the manuscript.

## Pre-publication history

The pre-publication history for this paper can be accessed here:

http://www.biomedcentral.com/1471-2458/10/129/prepub
